# The Effect of Cross-Linking Type on EPDM Elastomer Dynamics and Mechanical Properties: A Molecular Dynamics Simulation Study

**DOI:** 10.3390/polym14071308

**Published:** 2022-03-24

**Authors:** Yajian Wang, Huifang Liu, Pengpeng Li, Linbing Wang

**Affiliations:** 1 National Center for Materials Service Safety, University of Science and Technology Beijing (USTB), Beijing 100083, China; wangyj@ustb.edu.cn (Y.W.); b20200488@xs.ustb.edu.cn (H.L.); b202110537@xs.ustb.edu.cn (P.L.); 2 Joint USTB Virginia Tech Lab on Multifunctional Materials, University of Science and Technology Beijing (USTB), Beijing 100083, China; 3 Department Civil & Environmental Engineering, Virginia Tech, Blacksburg, VA 24061, USA

**Keywords:** EPDM, cross-linking, MD simulation, free volume, radius of gyration

## Abstract

The cross-linking structure of the Ethylene-propylene-diene monomer (EPDM) is made up of a number of cross-linking types, including carbon atoms from the main chain or monomer and ether crosslinks formed during degradation. Through molecular dynamic simulations, the contribution of each type of cross-linked structure to the dynamics and mechanical properties of EPDM, the study’s focus, were investigated. Cross-linking between the tertiary carbons of two main chains, cross-linking at the monomer’s unsaturated position, ether cross-linking after oxidation, and other combinations of target cross-linked carbon atoms from different positions, totaling eight types of cross-linked types, were mixed with EPDM free chains in a 1:1 ratio to form eight types of cross-linked EPDMs. These varieties of cross-linked EPDMs were then compared to an uncross-linked EPDM in terms of density, radius of gyration, free volume, mean square displacement, and uniaxial tensile stress-strain curves. It was found that the cross-linking was always proven to have a favorable influence on mechanical characteristics; however, the relaxation inhibition effect varied. The cross-linking between the diene monomer at the C9 position resulted in a more flexible molecular shape and was more than double the free volume of the uncross-linked EPDM, resulting in an improved diffusion ability. The ether cross-linking produced by the oxidation of the side chain cross-linking improved the positive contribution to stiffness and enhanced the inhibitory impact on diffusion properties, whereas the main chain cross-linking had the opposite effect. The research presented in this study leads to a better knowledge of the microscopic aspects underlying EPDM performance.

## 1. Introduction

A polymer presented of ethylene-propylene-diene monomer (EPDM) is commonly used for sealing, waterproofing, and vibration dampening [1,2,3,4]. It is frequently vulcanized or oxidized to generate a cross-linking structure and filled with carbon black materials to provide optimal qualities for certain applications [5]. Cross-linking is a covalent coupling technique that converts long-chain molecules’ viscous entanglement masses into three-dimensional elastic networks. To begin the oxidation process, sulfur vulcanization or a peroxide cure is used. Diene-monomers such as 5-ethylidene-2-norbornene (ENB), dicyclopentadiene, or 5-vinyl-2-norbonene are frequently used as reference places for oxidation [5]. EPDM, with its exceptional thermal aging resistance, is usually manufactured through peroxide-cured cross-linking, which can form carbon–carbon linkages [6]. Research into the details of such vulcanization mechanisms has been carried out by ^13^C NMR [7], while the atoms that make up these carbon–carbon crosslinks have been identified in both the side chains and the main chains [8].

Dicumyl peroxide (DCP) is the most often utilized peroxide cross-linking agent, and the thermal decomposition of the peroxide initiates the curing of EPDM [9]. EPDM macromolecular free radicals are formed as a result of the free radicals extracting hydrogen atoms from the EPDM polymer [10] and the tertiary carbon in the EPDM backbone has been proven to be a major source of hydrogen atoms [11,12]. On the one hand, they could combine to form a C-C crosslink between the tertiary carbons on polymer chains; on the other hand, they could participate in the remaining EPDM unsaturation with the resulting radicals undergoing hydrogen transfer from the C3 and C9 atoms of the ENB allyl, forming a crosslink between the two chains and also generating a new EPDM macromolecular radical poised for the next reaction [8,10]. As a product of the cross-linking of atoms at various places, multiple EPDM macromolecular chains with various conformations are formed. Ether cross-linking (C-O-C) has also been discovered in deteriorated and peroxided EPDM [2,13,14,15,16], and while only a single vulcanization method is utilized, numerous cross-linking forms are accessible in the EPDM system.

The influence of crosslink density on EPDM characteristics has been studied extensively in the past [6,17,18,19]; however, the impact of various kinds of cross-linking bonds on EPDM characteristics has yet to be investigated. This gap might be due to the difficulty of controlling EPDM cross-linking during manufacture. This research was made feasible by the advancement of molecular simulation tools. Thus, the goal of this work is to evaluate the influence of cross-linking types on the dynamical and mechanical characteristics of EPDM.

In the literature, a number of MD simulation studies on polymer characteristics have been carried out, which explore the kinetic properties of EPDM with the focus on the nature of transport, free volume, conformation, and glass transition [20,21,22]. Ma et al. investigated the thermal conductivity of EPDM networks using molecular dynamics modeling [23]. Wang et al. investigated the effects of products generated during aging including hydroxyl groups, chain scissions, and cross-linking on the structure and dynamics of EPDM [24]. Wang et al. constructed an EPDM cross-linking algorithm, whereby a cross-linked EPDM was established in the MD simulation process, and then they further investigated the free volume properties with the EPDM and the applicability of the principles of the time–temperature superposition principle (TTSP) and time–stress superposition principle (TSSP) [20]. Wang et al. used MD simulations to study the impact of thermal oxidation on an EPDM’s dynamic and mechanical characteristics [25], while previous investigations have confirmed the validity and the reliability of MD simulations for evaluating EPDM properties.

A series of MD simulations were carried out in this study to investigate the contribution of various cross-linked structures on the dynamics and mechanical properties of EPDM. The following outlines how the article is structured: (1) the possible cross-linked structures of ENB-EPDM during peroxide vulcanizations and degradations are performed, and the MD model ratios constituting the ENB-EPDM blends are presented; (2) the fundamentals of MD simulations are described, including the condensed-phase optimized molecular potentials for atomistic simulation studies (COMPASS) potentials, free volume calculations, periodic boundaries, radius of gyration, mean-squared displacement (MSD), and non-equilibrium-based stress-strain; (3) MD simulations were used to determine the density, radius of gyration (Rg), free volume, self-diffusion behavior, and stress-strain curves, which serve as proxies for the EPDM’s molecular conformation, relaxation, and mechanical characteristics, respectively; and (4) the findings and results of this work are summarized.

## 2. Cross-Linking Types of EPDM

Pure natural rubber is rarely cross-linked between molecular chains, and under load, the molecular chain segments are more prone to relative sliding, thus, the macroscopic properties show a significant viscosity. Although the long chains of molecules are often entangled with each other resulting in exhibiting some elasticity, this gradually disappears as the molecular chain segments are stretched. Therefore, in adapting natural rubber to industrial applications, various vulcanizing agents can be selected to create different cross-linked structures to produce a rubber with excellent properties, depending on the application.

### 2.1. Cross-Linking during Manufacturing

**Figure 1:**
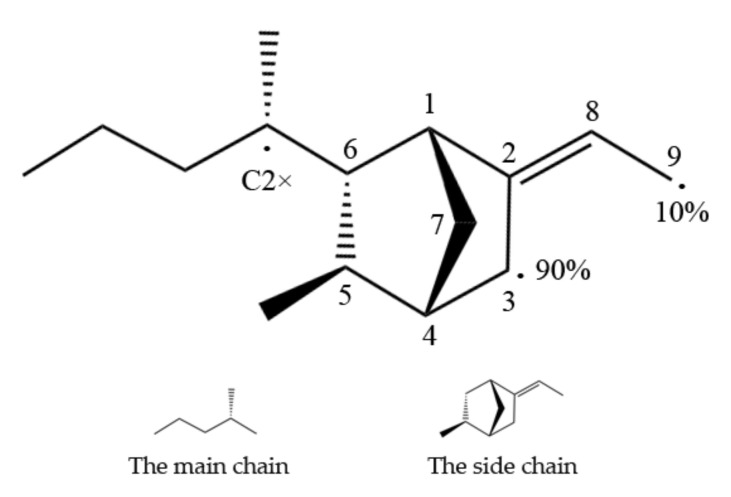
Possible EPDM reaction sites for the main chain and side chains (reprinted from [20]).

EPDM has a main chain and a side chain, as shown in Figure 1, and the main types of crosslinks formed are main–main chain cross-linking, main–side chain cross-linking, and side–side chain cross-linking. The target atoms for cross-linking are most likely the tertiary carbon(C2*) of the main chain and the C3 or C9 of the ENB monomer. The reaction rates of C3 and C9 were 90 % and 10%, respectively, according to earlier data [11].

**Figure 2:**
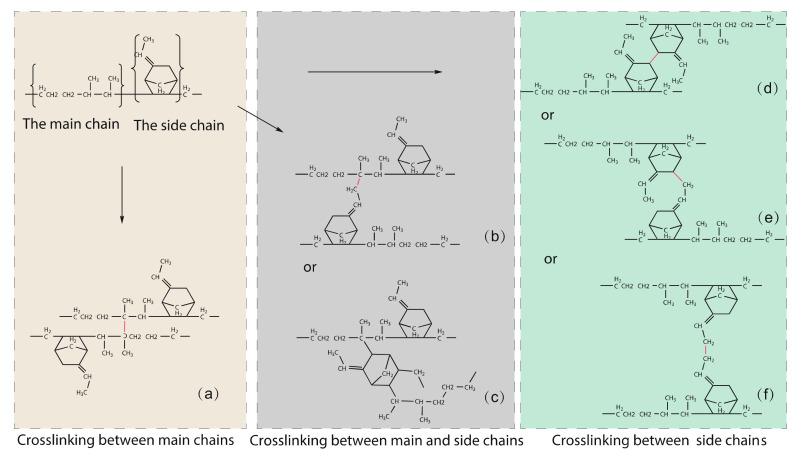
Types of cross-linking that are possible during the manufacturing of EPDM, where the cross-linking interaction is distinguished in red color. (**a**) is the C2*-C2* crosslinking, (**b**) is the C2*-C9 crosslinking, (**c**) is the C2*-C3 crosslinking, (**d**) is the C3-C3 crosslinking, (**e**) is the C3-C9 crosslinking, (**f**) is the C9-C9 crosslinking.

Figure 2 depicts the various forms of cross-linking that occur during EPDM manufacturing. The cross-linking between the two main chains is shown in Figure 2a, with the cross-linking point at the C2* atom. Figure 2b shows the cross-linking between the main chain and the side chain, with the target atoms being atom C2* of the main chain and atom C9 of the side chain, and Figure 2c shows the cross-linking between the main chain and the side chain, with the target atoms being atom C2* of the main chain and atom C3 of the side chain. The crosslinks between two side chains containing crosslinked atom pairs C3-C3, C3-C9, and C9-C9 are shown in Figure 2d,e,f, respectively.

### 2.2. Cross-Linking during Thermo-Oxidation

**Figure 3:**
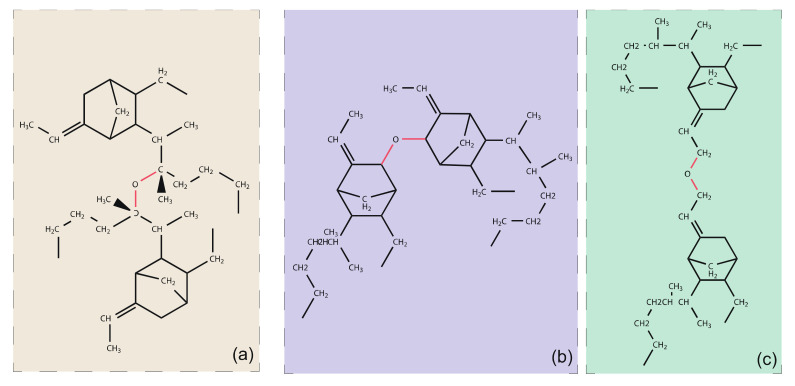
The possible regenerative cross-link architectures of EPDM after thermo-oxidation, (**a**) cross-linking between tertiary hydrogen carbons on the main chain, (**b**) cross-linking between two C3 atom on the side chains, (**c**) cross-linking between two C9 atoms on the side chains.

The products of thermal oxidation of EPDM are mainly ketones, carboxylic acids, hydroxyl alcohols, and ethers [2,25]. Of these, ethers are observed to be the main type of regenerative cross-link produced after thermal-oxidative ageing. These ethers may be grown in the main chain or in the side chain. The possible architectures are shown as Figure 3.

### 2.3. Construction of EPDM Cross-Linked Molecules

As per actual production ratios, 5 ethylene, 4 propylene and 1 ENB were copolymerized to form an EPDM unit, whose length was 26.526 Å. With this single chain, eight cross-linked conformations possibly resulting from the vulcanization or thermo-oxidation were built up.

**Figure 4:**
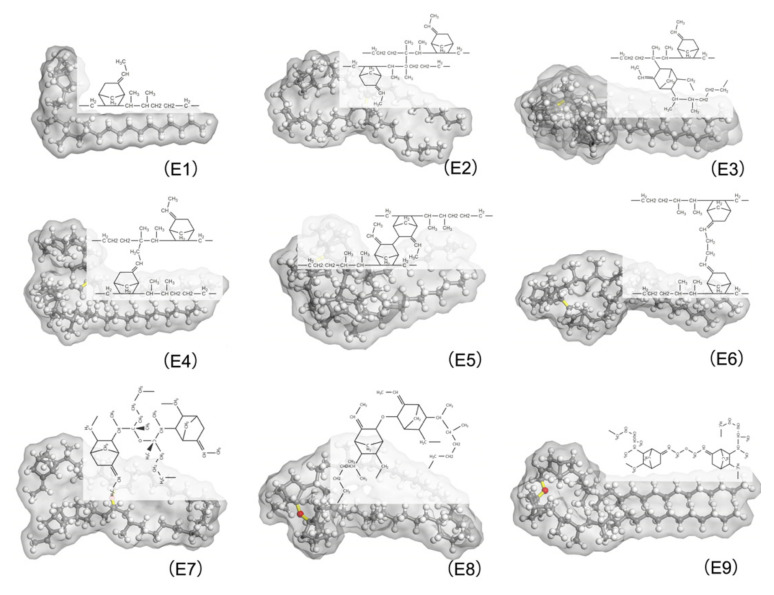
EPDM molecular chains with different cross-linking types, carbon atoms are shown in gray, hydrogen atoms in white, oxygen atoms in red, and cross-linking bonds in yellow. E1 was uncross-linked EPDM; E2, E3, E4, E5, E6, E7, E8, and E9 were EPDM with C2*-C2*, C2*-C3, C2-C9, C3-C3, C9-C9, C2*- O-C2*, C3-O-C3 and C9-O-C9 cross-links, respectively.

These eight types of molecules along with the EPDM free chain are shown in Figure 4. They are to be combined with EPDM free chains to form MD models representing various cross-linking EPDMs. The constituents of these MD models for the different cross-linking EPDM are shown in Table 1. M1 consisted entirely of E1. In addition to the 50% containing E1, the half components of M2, M3, M4, M5, M6, M7, M8, and M9 consisted of E2, E3, E4, E5, E6, E7, E8, and E9, respectively.

## 3. Molecular Dynamic Simulation Method

### 3.1. MD Simulations

The simulation work was performed in BIOVIA Materials Studio. Nine periodic MD models consisting of various types and numbers of cross-links (shown in Table 1) were constructed by employing the Amorphous Cell module. This was performed at a temperature of 298 k and a target density of 0.87 g/cm^3^, followed by 5000 steps of geometric optimization.

**Figure 5:**
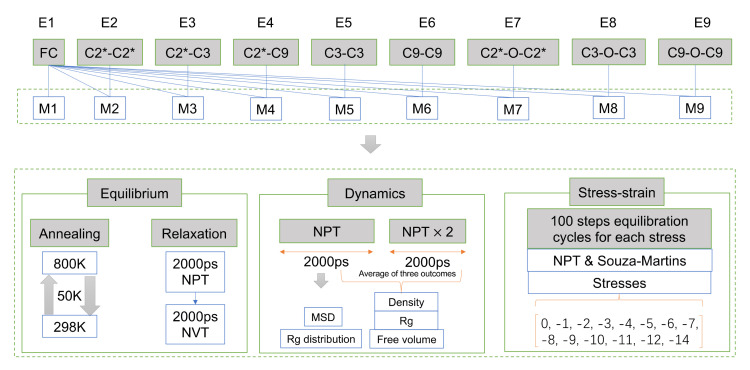
The workflow of molecular dynamics simulations in this study.

The MD simulation in this work were presented by the Forcite Package, and the whole process involved is shown in Figure 5. To produce a molecular conformation that more closely resembles the real EPDM material, the system needed to be optimally equilibrated, which was achieved by annealing and a series of kinetic equilibrium relaxations. The annealing was performed by heating the EPDM system sequentially from 298 K to 800 K with a temperature gradient of 50 K and cooled to 298 K with the same ramp. The 10 ps NPT dynamics were performed per ramp. The last frame of the annealed trajectory file was used as the initial structure for the kinetic equilibrium, and the system was successively subjected to NPT and NVT relaxation for 2000 ps under Berendsen thermostats. This was then followed by another 2000 ps NPT dynamics whose trajectory were used to compute the MSD and Rg distribution. The density evaluation, free volume and Rg were calculated here through averaging the results from three independent 2000 ps NPT simulations. The model closest to the mean values was used for the stress–strain calculations.

### 3.2. Potentials

The interaction potential function between individual particles (molecules or atoms) in a system and its associated parameters are known as the forcefield, which can be expressed as Equation (1):

(1) $$E_{total}=E_{vdW}+E_{coul}+E_{bond}+E_{angle}+E_{dihedral}+E_{invert}+E_{cross}$$

where *E_total_* is the total potential energy of the system, *E_vdW_* is the potential energy due to intermolecular forces, *E_coul_* is the Coulomb electrostatic potential, *E_bond_* is the bond extension energy, *E_angle_* is the bond angle potential, *E_dihedral_* is the dihedral rotation potential within the molecule, *E_inver_* is the off-plane vibrational potential and the *E_cross_* is the cross term.

The simulations of the conformational and thermophysical properties of EPDM in this paper were performed using the COMPASS force field [26].

The potential energy of the model can be expressed as Equations (2)–(8):

(2) $$E_{vdW}=\sum_{ij}\varepsilon_{ij}\left[2{\left(\frac{\sigma_{ij}}{r_{ij}}\right)}^{9}-3{\left(\frac{\sigma_{ij}}{r_{ij}}\right)}^{6}\right]$$

(3) $$E_{coul}=\sum_{elec}\frac{q_{i}q_{j}}{r_{ij}}$$

(4) $$E_{bonds}=\sum_{b}\left[k_{2}{\left(b-b_{0}\right)}^{2}+k_{3}{\left(b-b_{0}\right)}^{3}+k_{4}{\left(b-b_{0}\right)}^{4}\right]$$

(5) $$E_{angel}=\sum_{\theta }\left[k_{2}{\left(\theta -\theta_{0}\right)}^{2}+k_{3}{\left(\theta -\theta_{0}\right)}^{3}+k_{4}{\left(\theta -\theta_{0}\right)}^{4}\right]$$

(6) $$E_{dihedral}=\sum_{\chi }k_{2}\chi^{2}$$

(7) $$E_{invert}=\sum_{\phi }\left[k_{1}\left(1-\cos\phi \right)+k_{2}\left(1-\cos2\phi \right)+k_{3}\left(1-\cos3\phi \right)\right]$$

(8) $$\begin{array}{l} E_{cross}=\sum_{b,b^{\prime }}k\left(b-b_{0}\right)\left(b^{\prime }-b_{0}{}^{\prime }\right)+\sum_{b,\theta }k\left(b-b_{0}\right)\left(\theta -\theta_{0}\right)\\ +\sum_{b,\phi }k\left(b-b_{0}\right)\left(k_{1}\cos\phi +k_{2}\cos2\phi +k_{3}\cos3\phi \right)+\\ \sum_{\theta ,\phi }k\left(\theta -\theta_{0}\right)\left(k_{1}\cos\phi +k_{2}\cos2\phi +k_{3}\cos3\phi \right)+\\ \sum_{\theta ,\theta^{\prime }}k\left(\theta -\theta_{0}\right)\left(\theta^{\prime }-\theta_{0}{}^{\prime }\right)+\sum_{\theta ,\theta^{\prime },\phi }k\left(\theta -\theta_{0}\right)\left(\theta^{\prime }-\theta_{0}{}^{\prime }\right)\cos\phi \end{array}$$

where the subscripts *ij* and *elec* of the summation symbols denote the non-bonding potential energy from van der Waals and Coulomb, respectively; the bonding potentials are denoted by *bonds*, *angles* and *dihedral*; *b*_0_ and *θ*_0_ denote the equilibrium bond distance and angle value, respectively; and *k, k*_1_, *k*_2_, *k*_3_ are force constants. *r_ij_* represents the cut-off distance between atoms *i* and *j*; *ε_ij_* represents the potential energy parameter; *σ_ij_* denotes the finite distance at which the intermolecular potential energy becomes zero; and *q_i_* and *q_j_* are the fixed partial charges between atom *i* and *j* within the same molecule, respectively.

### 3.3. Periodic Boundary Conditions

**Figure 6:**
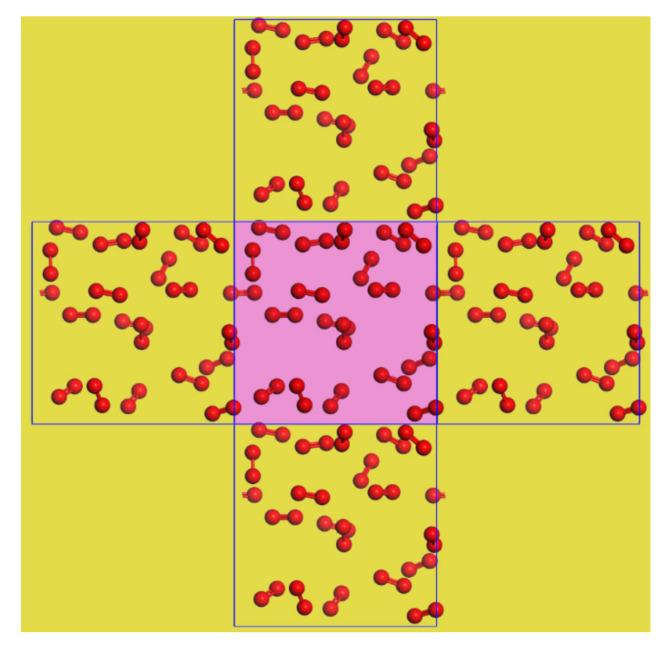
Schematic diagram of the periodic boundary of a planar system, with the simulated system in the center and the boundary enclosing its translated copies.

The calculation of the forces and potentials between the molecules or atoms in a system requires traversing all the atoms, which is the most computationally intensive portion of a molecular simulation. For a typical non-crystalline simulation, the molecular motion is highly stochastic, and a large fraction of the atoms are on the boundary of the model, where the force and potential fields are very different from those inside the system. The development of periodic boundary conditions perfectly addresses this issue. With periodic boundary conditions, the simulated system is in a space that is fully surrounded by its translated copies, forming an infinitely large lattice. As shown in Figure 6, when an atom moves to the initial box boundary, all boxes copy its path of motion. Thus, when an atom crosses one boundary of a box, it will appear with a same motion from the opposing side. To avoid duplication in the calculation of forces and potentials between the atoms in the mirror and atoms in the simulated system, the nearest mirror law is introduced, under which a particle in the simulated system only calculates the potential energy of the interaction between the particle closest to it.

### 3.4. Radius of Gyration

The radius of gyration (Rg), which is defined as the root mean square distance of each element of a body from its center of mass, is used here to describe the size of a macromolecule, its looseness, and the molecular conformation. It can be written as:

(9) $$\langle {\mathrm{Rg}}_{}^{2}\rangle =\frac{\sum {}_{i}m_{i}r_{i}^{2}}{\sum {}_{i}m_{i}}$$

where *m_i_* is the molecular chain’s mass and *r_i_* is the distance between the molecular chain and the center of mass.

### 3.5. Mean-Squared Displacement

The mobility of the molecules can somewhat reflect the viscosity and relaxation properties of the substance. In this paper, to compare the kinetic properties of EPDM consisting of different cross-links, we monitor the mean square displacement of particles during kinetic relaxation:

(10) $$\mathrm{MSD}=\langle {\left|r_{i}(t)-r_{i}(0)\right|}^{2}\rangle$$

where *r_i_*(*t*) denotes the position of the particle at moments *t* and *r_i_*(0) is the original position.

### 3.6. Uniaxial Stress–Strain Behavior

All elastic constants (canonical ensemble) and energies at uniform temperature can be calculated by the equilibrium method (microcanonical ensemble). Precision, on the other hand, necessitates a significant amount of computation time. The non-equilibrium approach is handy since it immediately applies pressure to the system while measuring strain. The elastic constants of the system are determined by applying multiple finite stresses and measuring the resulting strains, with consideration of the changes in entropy. This can be done by the Parrinello–Rahman or Souza–Martins barostat pressure control methods, which permit a variation of strain in the length and angle of atomic bonds or molecular chain segments to a limited extent [20,27]. The stress–strain curve was presented by applying increasing stresses in one direction (zero in the remaining directions) with 100 ps NPT equilibrium, then measuring the generated strain of the system.

### 3.7. Free Volume

**Figure 7:**
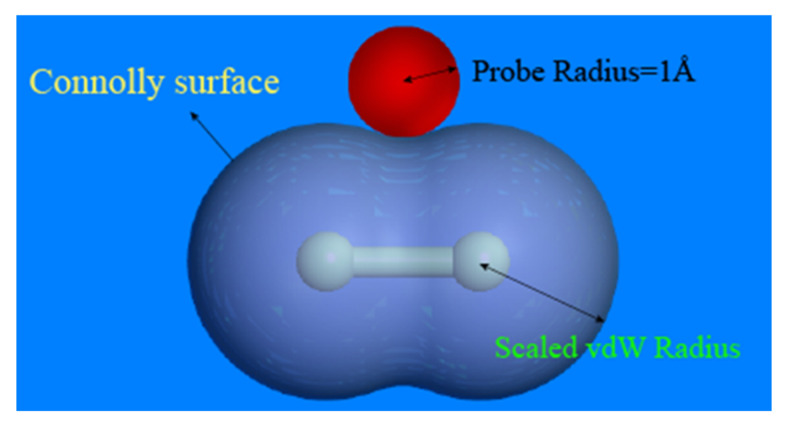
Connolly surface of chlorine molecule (reprinted from [21]).

The probe scan can be used to determine the EPDM model’s free volume. A Connolly surface defines the border between the probe and the atoms, as shown in Figure 7, with the free volume on the outside of the atomic surface and the occupied volume on the inside [28]. In this paper, the model’s free volume was estimated by generating a Connolly surface and splitting the periodic bounding box into multiple grids with 0.15 Å spacing. The free volume can be calculated by scanning these grids with a probe of radius 1 Å. If over half of the grid is occupied by the probe, it will be marked as “free”, and if more than one half of the grid is filled with atoms, it will be marked as “occupied”. When two unoccupied grids are adjacent, they are recognized as a free volume and the proportion of the free volume to the total volume is defined as the fractional free volume (FFV) [29].

## 4. Results and Discussion

### 4.1. Relaxation

Constrained by computing resources, only one of the systems was allowed to run for 10 ns, whose results were compared with the 2 ns simulations as a way of verifying if the proposed running times allowed the system to converge properly.

**Figure 8:**
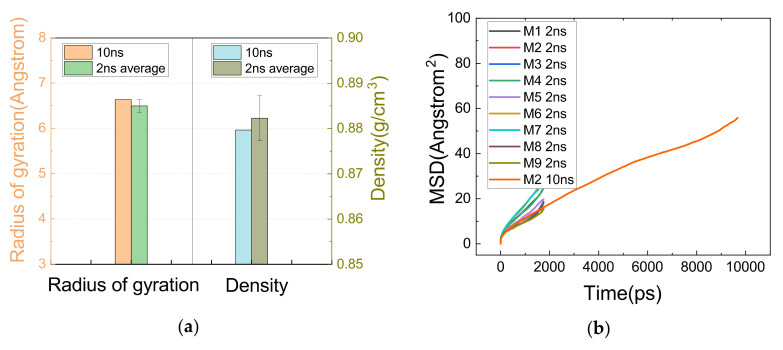
Comparison of results for 10 ns and 2 ns simulations. (**a**) Comparison of density and Rg between 10 ns and 2 ns simulations. (**b**) MSD results for 10 ns and 2 ns.

As can be seen in Figure 8a, both the deviation of Rg and density between the 10 ns and 2 ns results was within the error range of 2 ns calculations, and 8b shows that the slopes of the MSD curves obtained from the 10 ns and 2 ns simulations were in good agreement. Thus, it is roughly considered that the system was properly relaxed.

### 4.2. Density

**Figure 9:**
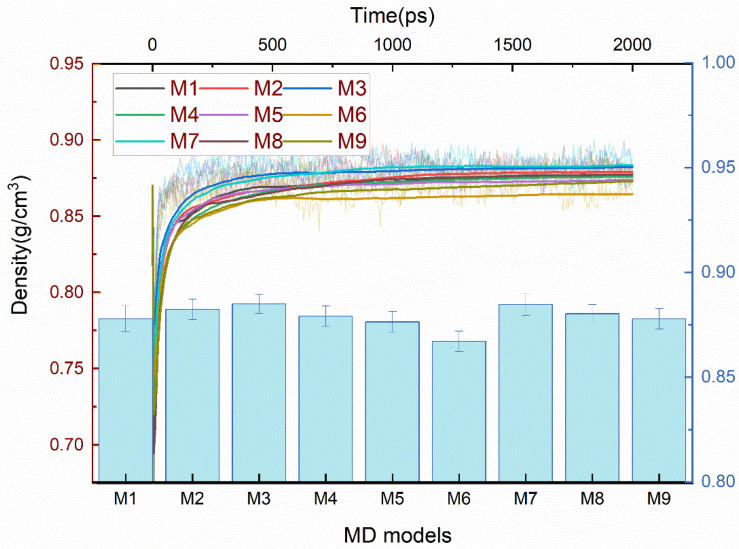
The density of varied cross-linked EPDM MD models. The fluctuation curves are the density evolution of 2000 ps NPT, the smoothed curves are the average density of the kinetic process, and the histogram is the average of the three independent MD models.

As shown in Figure 9, the M3 with C2*-C3 cross-links and M7 with C2*-O-C2* cross-links were denser than the others, and the M2 with C2*-C2* cross-linking, M4 with C2*-C9, and M8 with C3-O-C3 cross-linking were denser than the M1 without cross-links, M5 with C3-C3, and M9 with C9-O-C9 cross-linking. The M6 with C9-C9 cross-links were less dense than the above models. Of these reactants and products, the lowest density occurred at the cross-linking bonding of C9-C9, and it can be verified from the results that the M9 was less dense than the M7 and M8, and that the M6 was less dense than the M2 and M5. This might be related to the configuration of the cross-linked structure and the volume created, which will be explained in the subsequent analysis.

### 4.3. Radical Radius

**Figure 10:**
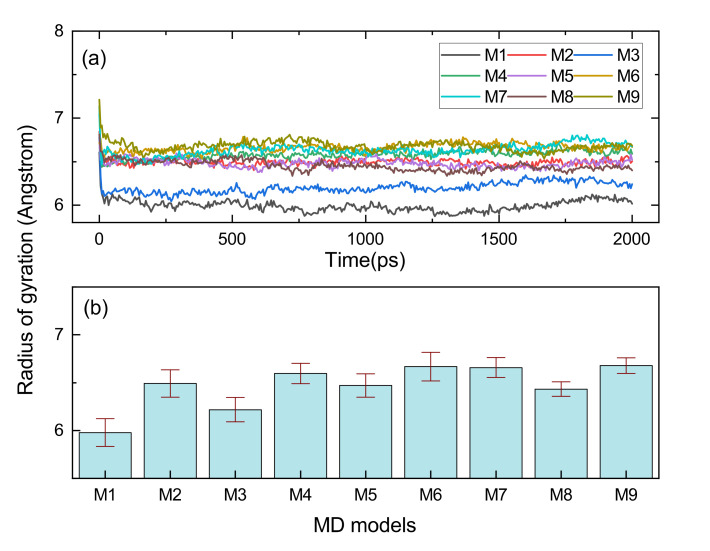
The Rg variation monitoring results when performing 2000 ps NPT MD simulation for EPDM models. (**a**) is the Rg evolution curve, (**b**) is the b is the Rg average value of three independent NPT simulations with standard deviation.

**Figure 11:**
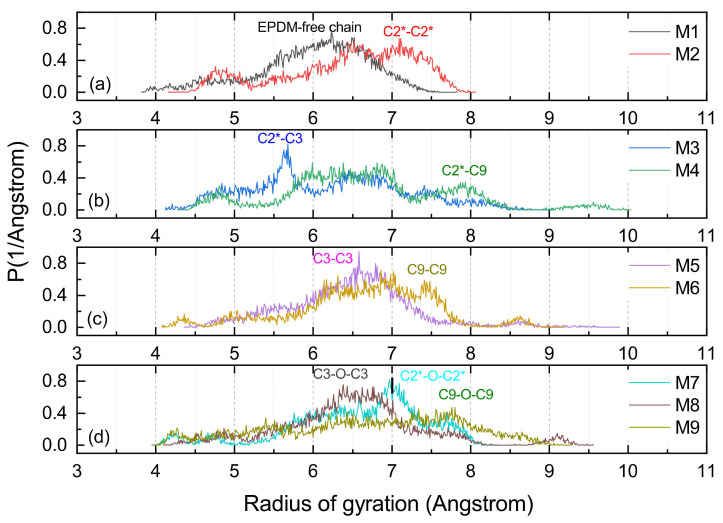
The Rg distribution of varied cross-linking EPDM MD models. (**a**) shows the results for M1 and M2, (**b**) is for M3 and M4, (**c**) is for M5 and M6, (**d**) is for M7, M8, and M9.

Rg is suitable for a proper assessment of the volume shaped by the molecular chain, and the distribution function of the Rg could serve to evaluate the conformational flexibility of long chains [30,31]. As shown in Figure 10, all the MD models with cross-links had a larger Rg than the M1, and this is because cross-linking commonly causes a large radical radius. The structure shaped by C9-O-C9 had the largest Rg of any of the cross-linking types. Furthermore, it could be found that the Rg of M6 (C9-C9) and M4 (C2*-C9) were at a high level, which can be verified from Figure 11b–d, and the cross-linking types with C9 consisted of C2*-C9, C9-O-C9 and C9-C9 with a radius of gyration distribution between 7.5 and 9 Å. The large span and flattened peaks of the Rg distribution curves for the M4 and M6 imply that their molecular segments were more flexible.

The cross-links between the main chains involving the M2 with C2*-C2* and M7 with C2*-O-C2* shape a larger Rg than the cross-links of M3 (C2*-C3) and M8 (C3-O-C3) in which the atom C3 is participating. Figure 11d shows that the radius of gyration of the M7 had a characteristic peak between 7 and 8 Å, implying that it was susceptible to molecular entanglement and that it was easy to produce strong topological constraints.

### 4.4. Free Volume

**Figure 12:**
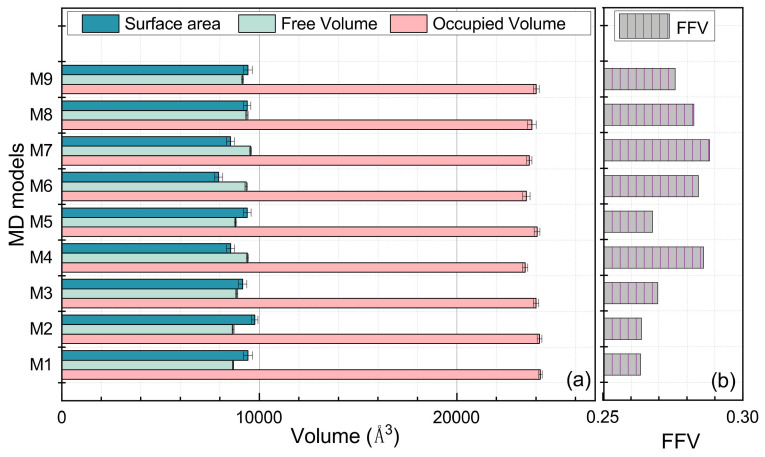
The volumetric parameters of the MD models. (**a**) describes the occupied volume, the free volume and the area of the Connolly surface of varied cross-linked EPDM models, and (**b**) illustrates the FFV of these MD models.

As illustrated in Figure 12, the M1 model, which was composed entirely of free chains, had the largest occupied volume. The occupied volume for the cross-linking EPDM models in descending order was: M2 > M5 > M9 > M4 > M8 > M7 > M6 > M3. The M1 free volume was the smallest, which can be attributed to the smallest radius of gyration created by the uncross-linked structure. The free volume of cross-linked structures in descending order was: M7 > M8 > M3 > M6 > M9 > M4 > M5 > M2. The Connolly surface area in descending order was: M2 > M9 > M8 > M5 > M1 > M4 > M3 > M7 > M6. The free volume fractions of these EPDM models in descending order were: M7 > M3 > M6 > M8 > M9 > M4 > M5 > M2 > M1, with M1 also having the smallest FFV.

### 4.5. Diffusion

**Figure 13:**
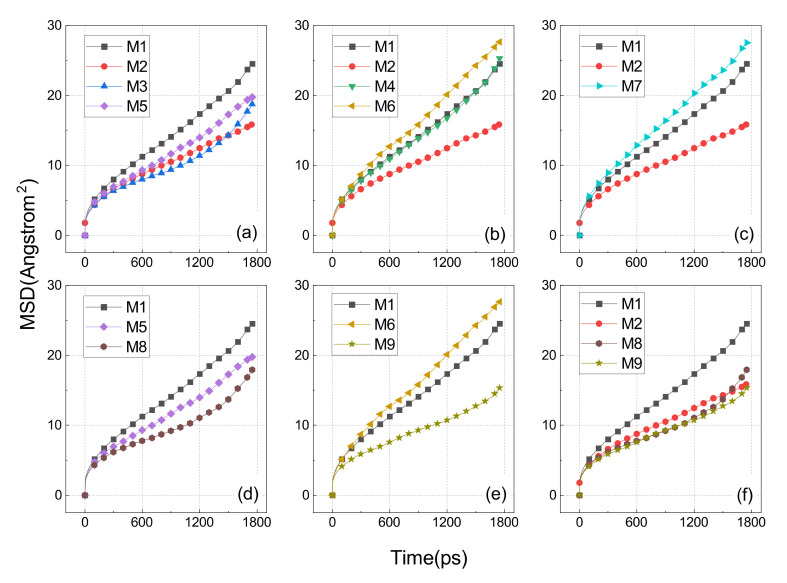
MSD of various cross-linked EPDM. (**a**) compares the cross-linking involving the C3 atom of the side chain; (**b**) compares the cross-linking involving the C9 atom of the side chain; (**c**) demonstrates the effect of oxidation on the cross-linking between the tertiary hydrogen carbons of the main chain; (**d**) shows the impact of oxidation on the cross-linking between the C3 atoms sitting on two side chains; (**e**) illustrates the effect of oxidation on the cross-linking between the C9 of the two side chains; and (**f**) is a comparison showing the two aged side chain–side chain cross-links.

The slope of the MSD curve indicates the self-diffusion coefficient of the broken molecular chain. A large slope indicates a molecular structure with good motility, meaning that the molecules are less constrained and more prone to creep and relaxation. To minimize the statistical errors, we selected the first 1800 s of 2000 ps for the MSD calculations, which are displayed in Figure 13.

Figure 13a shows the comparison of MSD results for the M1, M2, M3, and M5 models which represent uncross-linked EPDM, the main chain cross-linking EPDM, main–side cross-linking EPDM at atom C3, and side chain cross-linking EPDM at atom C3, respectively. The order of slope from largest to smallest was: M1 > M5 > M3 > M2, which means that the cross-linking interaction between the main chain C2-C2 was stronger than the cross-linking interaction between the main chain and the C3 located at the side chain, while the cross-linking between the two C3 atoms sitting on two side chains was the weakest of them all.

The comparative results shown in Figure 13b illustrate the effect of side chain cross-linking at the C9 atom. As seen in the graph, the slopes were M6, M4, M1, M2 in order ascending. The cross-linked molecular structures arising at the C9 atom were instead more mobile than the uncross-linked ones (i.e., M1). One of the reasons for this can be attributed to a more flexible molecular chain for the C9-C9 and C2-C9, that is a result of the weaker entanglement between the molecular chains of such cross-links. The other reason could also be ascribed to the large free volume created by such cross-links, providing more space for chain movement.

The reasonableness of the second explanation above can be further verified in Figure 13c, where the slope of M7 was slightly greater than that of M1. This is because the large FFV of M7 dominates its relaxed nature, although it had a pronounced peak in the radius of gyration distribution (as shown in Figure 11d). M7 could be regarded as an oxidation product of M2, which is also consistent with the phenomenon of rubber getting softer as it ages.

As shown in Figure 13d, the slopes of M5 and M8 were both smaller than M1, and M8 was slightly smaller than M5. This means that the side–side cross-link established at the C3 atom of two side chains has a slight reduction in its molecular motility after ageing.

Figure 13e demonstrates the effect of ageing on the cross-link established between the C9 atoms of two side chains. The FFV of M6 was more than twice that of M1, and the molecular segments of E6 were more flexible, so that the M6, despite its cross-linking, was instead more mobile than the M1.

The results in Figure 13f show a comparison of three types of cross-links that constrain the motion ability of the molecules. The slopes of M2 and M9 were almost identical and the slope of M8 was slightly larger than them, which means that the C9-O-C9 and C2-C2 were almost consistent in their constraining effect on the molecular motion. The FFV of M8 and M9 was more than twice that of M2, which somehow indicates that the combination effect of the cross-linking and entanglement of M9 and M8 was stronger than that of the M2.

### 4.6. Uniaxial Tensile Properties

**Figure 14:**
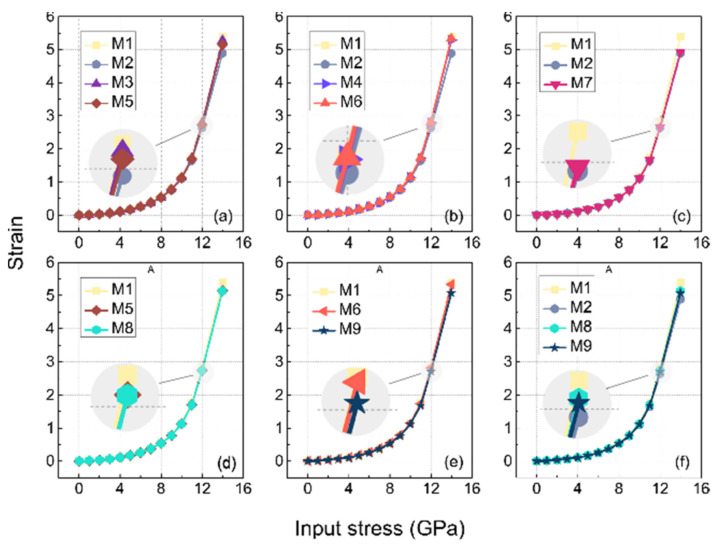
Uniaxial tensile stress–strain curve of EPDM models with different cross-linking, (**a**–**f**) are drawn for the same purpose as in Figure 13.

In practice, uniaxial tensile properties can respond to the strength of the cross-linking, especially in the case of finite strains, where the cross-linked bond is an important element present to avoid shear failure of the structure. In MD simulations, changes in the stiffness of a material are better explained by entropy than by internal energy. In this paper, it is roughly defined as the system with large strains after applying equal stresses, and the conformational entropy of the system with large strain is larger compared to those with smaller strains [32]. Due to the size of the model in this study, the difference in the macroscopic properties of a model constructed from microstructures with differences was difficult to clearly observe, but it was subject to regular variation. Here we make a partial zoom of the image to Figure 14 to slightly ameliorate this problem.

As shown in Figure 14, the strain in M1 was the largest under the input stress of 12 GPa among all the subplots because it was not cross-linked. The strains in the descending order after M1 were M3, M5, and M2 in Figure 14a, which reveals that the conformational entropy between the side chains (C3-C3) was lower than the cross-linking of the side and main chains (C2*-C3) in the type of cross-linking involving C3 atoms, while their conformational entropies were both stronger than the cross-linking between the main chains (C2*-C2*). This finding is also corroborated in the MSD results, where the slope of the MSD curve of the C2*-C2* were smaller than the other cross-links.

The strain magnitude of M6 and M4 in Figure 14b were almost equal to that of the M1, which suggests that the conformational entropy generated at the C9 atom of the side chain (i.e., C9-C9 cross-linking, and C2*-C9) was larger, compared to the cross-linking of the main chain (C2*-C2*).

Figure 14c shows that the strain magnitude of the M7 was slightly greater than that of the M2, which indicates that the entropy of ether cross-linking resulting from the oxidation of the origin cross-linking (C2*-C2*) increased. Compared with other types of cross-linking, the cross-linking between the main chains had a more significant increase in modulus.

From Figure 14d, it can be seen that the strain amplitudes of M8 and M4 almost coincided, and it can be concluded that the contribution of the C3-C3 cross-linking to the mechanical modulus remained little changed after oxidation. A similar observation was found in the MSD results (as shown in Figure 13d).

It can be seen in Figure 14e that the strain amplitude of the M9 was larger than that of the M6, and it can be inferred that the contribution of the C9-C9 crosslinks to the modulus increased after oxidation. Combining Figure 14f and Figure 14c, we found that the strain levels were, in descending order, M2, M7, M9, M8 at an input stress of 12 Gpa, which means that the cross-linking between the main chains contributed more to the modulus than the cross-linking between the side chains, both before and after oxidation.

In conclusion, the conformational entropy of cross-linking between the main chains contributed much to the modulus, and that of the cross-linking between the C9 atoms of the side chains was larger than the C3 atoms of side chains. The entropy of the cross-linking constructed by the side and the main chain was between that of the C9-C9 and C3-C3. All the entropy of the side chain cross-linking that was decreased by oxidation, especially for the C9-C9, was significant.

## 5. Conclusions

EPDM is widely utilized as a sealing and vibration damping material, and its cross-linking interaction has a strong influence on its performance. EPDM has various different types of cross-linking that can occur in the main chain, side chain, or during thermal oxidation to generate regenerative ether cross-linking with oxygen. This paper’s contribution was to look into the impact of probable cross-linkages on an EPDM’s dynamical and mechanical properties. To provide it, eight cross-linked EPDM molecular structures that may occur during manufacture and thermal oxidation were generated, and each was combined 1:1 with EPDM free chains to build MD models representing distinct cross-linking EPDM. The density, free volume, radius of gyration, mean square displacement, and uniaxial tensile stress–strain calculations were then performed on these MD models. On the basis of comparisons with uncross-linked EPDM, the contribution of each cross-link type to the kinetic and mechanical properties of EPDM was evaluated qualitatively. The following are the key findings of this research:

(1) The Rg shaped by cross-linked bonds with C9 participation is greater than C2*, the Rg shaped with C3 participation is smaller, and the free volume associated with the Rg follows this pattern as well. In comparison to the origin cross-linking, the Rg shaped by oxidized cross-linking of C2*-O-C2* and C9-O-C9 grows greater, whereas that of C3-O-C3 becomes smaller.
(2) According to the models developed in this paper, cross-linking of C2*-C2*, C3-O-C3, and C9-O-C9 has a significant constraining effect on molecular diffusion motion, C2*-C3, and C3-C3 have a minor inhibiting effect on diffusion, while C2*-C9 and C9-C9 boost molecular self-diffusion motion.
(3) The topological restrictions are weaker in C2*-C9 and C9-C9, and the FFV of M4 and M9 is more than double that of M1, offering greater free space for chain movement, which is why M4 and M6 have superior self-diffusion ability while being more cross-linked than M1.
(4) Cross-links between the main chains significantly provide a positive contribution to the modulus, and the conformational entropy of the cross-linking between the C9 atoms of the side chains is higher than that between the C3 atoms. The effect of cross-links made up of the side chains and the main chain have an entropy in between that of C9-C9 and C3-C3.
(5) The ether cross-linking formed by the oxidation of the side chain cross-linking enhances the inhibition effect on diffusion properties while the main chain cross-linking has the inverse result.

## Figures and Tables

**Table 1:** Composition and naming of the cross-linked MD model of EPDM.

	Molecular Types	M1	M2	M3	M4	M5	M6	M7	M8	M9
E1	Free chain	40	20	20	20	20	20	20	20	20
E2	C2*-C2*		10							
E3	C2*-C3			10						
E4	C2*-C9				10					
E5	C3-C3					10				
E6	C9-C9						10			
E7	C2*-O-C2*							10		
E8	C3-O-C3								10	
E9	C9-O-C9									10

## Data Availability

Data is contained within the article.
